# 

**Published:** 1868-03-01

**Authors:** 


### CONTENTS:

Physiology. Investigations, etc., (continued.) By Walter Hay, M.D.... 155

Clinical Cases.... 165

Hygienic Treatment of Patients. By J. Little, M.D., LeRoy, Ill.... 166

Cases in Practice. By W. Anderson, M.D., LeRoy, Ill.... 176

Carcinoma of the Stomach. By J. W. Dora, M.D., Mattoon, Ill.... 177

BOOK NOTICES:

Observations on the Nature and Treatment of Polypus of the Ear. By Edward H. Clark, M.D., Prof., etc.... 180

EDITORIAL:

Back Numbers.... 181

Circular.... 181

Advertising Specialties.... 182

Bliss & Sharp.... 183

Duns.... 183

Paris Items.... 184

LOOT:

Iodine and Carbolic Acid.... 185

Gentian Root as a Dilator.... 185

